# Bono Bo and Fla Mingo: Reflections of Speech Prosody in German Second Graders’ Writing to Dictation

**DOI:** 10.3389/fpsyg.2016.00856

**Published:** 2016-06-10

**Authors:** Frank Domahs, Katharina Blessing, Christina Kauschke, Ulrike Domahs

**Affiliations:** ^1^Institute of Germanic Linguistics, Philipps University MarburgMarburg, Germany; ^2^Faculty of Education, Free University of Bozen-BolzanoBozen-Bolzano, Italy

**Keywords:** writing acquisition, foot, syllable, graphemic word, prosody, orthography

## Abstract

Written German is characterized by an underrepresentation of prosody. During writing acquisition, children have to tackle the question which prosodic features are realized by what means – if any. We examined traces of speech prosody in German children’s writing to dictation. A sample of 79 second graders were asked to write down eight sentences to dictation. We analyzed three potential reflections of speech prosody in children’s dictations: (a) Merging of preposition and definite article, potentially preferred after monosyllabic prepositions as in this case preposition and article may melt to the canonical trochaic foot in German. (b) The introduction of orthographically inadequate graphemic border markings within trisyllabic animal names, respecting borders of prosodic units like foot or syllable. (c) Omissions of the definite article in non-optimal prosodic positions, deviating from the preferred strong-weak rhythm. The occurrence of border markings was evaluated via graded perceptual judgments. We found no evidence for inter-word border markings being influenced by prosodic context, probably due to a ceiling effect. However, word-internal markings within animal names, although rarely occurring in general, were clearly influenced by prosodic structure: Most of them were produced at borders of feet or syllables, while significantly fewer markings were perceived at borders of syllable constituents or within consonant clusters. Moreover, we observed significantly more omissions of the definite article in non-optimal prosodic positions compared to potentially optimal positions. Thus, our results provide first evidence from writing acquisition for prosodic influences on writing in a language with scarce graphemic marking of prosody.

## Introduction

During writing acquisition, children have to tackle the question which prosodic entities and features of oral speech are realized orthographically by what means – if any. Most of those prosodic features and entities are typically not object of overt formal instruction. Thus, schoolchildren have to find out themselves how prosody is realized in written language. In the present study we used non-conventional spellings (i.e., ‘errors’) in second graders’ writing to dictation to investigate systematic reflections of speech prosody in writing acquisition.

In the following parts of the Introduction, we will first review prosodic features of written German. Afterward we will summarize evidence on children’s sensitivity to relevant prosodic cues and entities in spoken German. Finally, we will sketch the acquisition of graphemic borders.

### Prosody in Written German

Written German, as well as other writing systems, is characterized by an underrepresentation of prosody (Treiman and Kessler, 2014), i.e., explicit marking of prosodic entities like syllables, feet, and prosodic words may either be non-salient, ambiguous, or even completely lacking. In the following, we will discuss graphemic markers for words, syllables, feet, stress, and rhythm.

Clearest markers are indicative of phonological words, as far as they happen to co-occur with lexical, morphological, or syntactic^1^ words (Jacobs, 2005; Fuhrhop, 2008; Evertz, 2016). They are unambiguously delimited by spaces, sometimes combined with capitalization of the initial letter (e.g., for nouns and proper names) or punctuation. However, if borders of phonological words do not converge with those of lexical words, markers follow lexical rather than prosodic principles, such that one phonological word may be spelled as two graphemic words (e.g., <siehst du> rather than ^∗^<siehstu> [Engl. lit. see you])^2^ or two phonological words may be realized as one graphemic word (e.g., <Langeweile> rather than ^∗^<Lange Weile> [boredom]). In this respect, German differs from other languages as Spanish or Arabic (Treiman and Kessler, 2014). As in spoken language, German graphemic words should at least consist of one foot which should consist of at least one full syllable (Fuhrhop, 2008; Evertz, 2016).

In German, as in other written languages using the Latin alphabet, syllables are marked by a combination of probabilistic cues, including graphemic length and graphotactic probability. First, syllabic nuclei are always realized by compact (vowel) letters without ascenders or descenders (e.g., <a>, <e>, <i>, <o>, <u>), while (consonant) letters with or without ascenders or descenders (e.g., <f>, <k>, <p> or <m>, <n>, <r>) may fill onset and/or coda positions (Naumann, 1989; Primus, 2003; Fuhrhop, 2008; Evertz and Primus, 2013; Evertz, 2016). In this way, graphemic syllables are characterized by a U-curved graphemic length distribution, where length maxima mark syllabic boundaries and the length minimum marks the syllabic nucleus. However, the relationship between length and position is only probabilistic in nature (for a discussion of relevant exceptions see Fuhrhop and Buchmann, 2016). Second, as in spoken language, syllabic borders are marked by phonotactic/graphotactic probabilities (Chetail and Mathey, 2009; Chetail and Content, 2012a; Chetail et al., 2014). Although syllabification in spoken and written language often lead to analogous results, they may also diverge (Chetail and Content, 2012b).

In analogy to spoken language, syllables in written language can be part of graphemic feet. In German (as in English), canonical feet are trochaic, either monosyllabic or bisyllabic with a final reduction syllable (Evertz and Primus, 2013; Evertz, 2016). In short, graphemic reduction syllables are characterized by the nature of their nucleus (always <e>) and the absence of complex margins. Thus, <Rabe> and <Robbe> [raven, seal] would form canonical graphemic (as well as phonological) feet. Non-canonical feet are bisyllabic without reduction syllable. Note, that – according to this definition – trisyllabic words like <Bonobo>, <Giraffe>, or <Papagei> [bonobo, giraffe, parrot] exceed the range of canonical graphemic feet.

Canonical graphemic feet are transparently related to word stress: In monosyllabic feet, there is no choice where to place stress, while in canonical bisyllabic feet only the first (i.e., the only full) syllable can bear stress. Stress assignment is more complicated and largely opaque in words with non-canonical feet or in polysyllabic words. This leads to a general need for lexical specification of stress in German. However, beyond lexicalization there is accumulating evidence for a role of syllable weight in stress assignment, such that light final and closed prefinal syllables are associated with penultimate stress whereas (super) heavy final syllables, especially if combined with light prefinal syllables, lead to stress on the antepenultimate or ultimate syllable (Janßen, 2003; Janßen and Domahs, 2008; Röttger et al., 2012; Domahs et al., 2014). Graphemic structure has an influence which is at least partly independent from phonological structure as can be seen in the case of complex graphemes which consist of two or three letters but correspond to only one phoneme (e.g., <sch>, <ch>). It has been shown that such complex graphemes modulate stress assignment over and above their phonemic value (Röttger et al., 2012). In any case, the main stress position indicates the strong syllable of a foot. In case of bisyllabic feet, the stressed syllable is always the left one.

To some extent, written German also reflects rhythmic properties of the spoken language. This becomes apparent in sequences of bisyllabic trochees, where pairs of full and reduced graphemic syllables are regularly separated by spaces. However, lexical and syntactic principles prevail again, such that two lexical words should not be written together in one graphemic word (e.g., <in den> rather than ^∗^<inden> [into the]) or lexical words should not be separated graphemically (e.g., <Krokodil in> rather than ^∗^<Kroko dilin> [crocodile into]) to bring rhythmic structure to the surface. Interestingly, a specific property of written German yields some leveling of syllabic length. A variant of <h> which itself is silent but indicates vowel lengthening is more likely to be used in otherwise short syllables (e.g., <Ahn>, <Lahn> [ancestor, river Lahn]), but not in graphemic syllables which are already long and thus ‘visually heavy’ (e.g., <Schwan> rather than ^∗^<Schwahn>[swan]) (Eisenberg, 2000). Similarly, vowel doubling preferably takes place in relatively short (e.g., <Boot> [boat]) rather than in visually more complex syllables (e.g., <Brot> or <Schrot> rather than ^∗^<Broot> or ^∗^<Schroot> [bread, whole grain]). In this way, length leveling of graphemic syllables may lead to a more regular rhythmic surface structure in written German, as between-syllable distances become less heterogeneous. Although not explicitly taught at school, this property of the German writing system seems to be acquired by competent writers (Domahs et al., 2001).

In sum, different features or units of speech prosody are marked by different orthographic means and with different degrees of cue validity. While words are marked consistently by spaces (and, if applicable, by capitalization), other features or units are marked by more probabilistic cues related to the form of letters or syllables, but not by spaces or capitals. A graphemic word contains at least one graphemic foot, which contains at least one graphemic syllable. Bisyllabic canonical feet consist of a full and a reduced syllable. However, trisyllabic words exceed the size of canonical feet. Rhythm may surface as a side-product of the most frequent foot type and syllabic length leveling, but in doubt, lexico-syntactic rules overwrite prosody in written German. Obviously, it is a challenging task in writing acquisition to learn these regularities.

### Sensitivity to Prosodic Phenomena in Spoken Language Development

Before we will turn to the acquisition of graphemic borders, it seems worthwhile to point out that children in second grade (or even considerably earlier) are well sensitive to the relevant prosodic phenomena in spoken language.

Already in the first year of life, infants are able to segment linguistic units (e.g., words or phrases) out of the continuous stream of speech (Demuth, 2009). To this end, they use the predominant prosodic pattern (i.e., the trochee in German), which they already prefer over the non-dominant pattern by the age of 6 months (Jusczyk et al., 1999; Höhle et al., 2009). Moreover, from about 9 months on, infants are able to detect phonotactic probabilities which indicate syllable onsets and to use them for speech segmentation and lexical acquisition (Mattys et al., 1999; Coady and Aslin, 2004; Friedrich and Friederici, 2005). At about 8 months infants are able to recognize even unstressed high frequent content and function words within the speech stream (Höhle and Weissenborn, 2003).

There is also sound evidence for a role of prosodic feet in children’s speech processing. In particular, it has been shown repeatedly that young children omit unstressed unfooted syllables in speech repetition, affecting both function words (McGregor and Leonard, 1994; Wijnen et al., 1994; Gerken, 1996; Boyle and Gerken, 1997) and unstressed unfooted syllables in polysyllabic words (Klein, 1981; Gerken, 1994; Carter and Gerken, 2004), although there is evidence suggesting that the omitted syllables are indeed perceived and mentally represented (Wijnen et al., 1994; Carter and Gerken, 2004). The probability of weak syllable omissions is further modulated by the attachment of these syllables to phonological words and phrases (Gerken, 1996). By and large, prosodic development at the word level can be regarded as accomplished by the age of two and a half years (Grimm, 2010; Kauschke, 2012).

The optimal prosodic structure of phrases and sentences in German and other languages builds up a strong-weak rhythm (Liberman and Prince, 1977; Vogel et al., 2015). Although violations of this *Rhythm Rule* do occur frequently in spoken German, they still have behavioral as well as electrophysiological consequences (e.g., Bohn et al., 2013). Young children do not only use rhythmical regularities for word segmentation in speech perception, but also in their strategies of word sequencing in speech production (McDonald et al., 1993; Vogel et al., 2015 for adults; Franz, 2015, for preschool children).

To conclude, typically developing children at an age of 7 or 8 years, as tested in the present study, are sensitive to prosodic structures in spoken language like word, phrase, syllable, subsyllabic constituent, foot, stress, or rhythm. Of course, some interindividual heterogeneity of prosodic representations and/or performance exists and may persist into school age or even adulthood (Haake et al., 2013; Heisterueber et al., 2014).

### Graphemic Borders in the Development of Writing

During writing acquisition, children are typically not explicitly taught which borders are to be marked and how at the word or sub-word level (Bredel, 2006). Nevertheless, the principle of word-based border marking seems to be acquired quite robustly such that at the end of this development <1 per cent of all adult writing errors is related to border marking (Bredel, 2006).

The acquisition process passes through several developmental stages, some of which may overlap or be skipped in individual children (Bredel, 2006). In the following, we will sketch developmental stages according to Bredel (2006) which apply to children who are already phonological spellers (Treiman and Kessler, 2014). At the first stage, children start to write in ‘*scriptio continua’*, i.e., without any explicit border marking (see Bredel, 2006 for German; de Gòes and Martlew, 1983, for English; Sandbank, 2001, for Hebrew). Writing at the second stage is mainly guided by speech prosody. This may manifest itself in highlighting stressed syllables, for instance by capitalization (e.g., ^∗^<eDe> instead of <Idee> [idea] or ^∗^<zuGrozmta> instead of <zu Großmutter> [to grandmother], Jakob, 6 years, reported by Weinhold, 2000, as cited in Bredel, 2006), in orthographically deviant merging of two lexical words into one (e.g., ^∗^<FESCH|SECH> instead of <wäscht sich> [washes himself] or ^∗^<WENK|DEN> instead of <wringt den> [wrings the], Robert, 8 years, reported by Röber-Siekmeyer, 1998, as cited in Bredel, 2006, ‘|’ denoting an omitted border), or in orthographically deviant separations of one word into two parts (e.g., ^∗^<ge^∧^komen> instead of <gekommen> [come] or ^∗^<ge^∧^flogen> instead of <geflogen> [flown], Alexander, first grade, reported by Bredel, 2006, ‘^∧^’ denoting an added border marking). In general, merging, i.e., failure to separate two or more written words with a space, seems to be more common than hypersegmentation, i.e., splitting one target word into more than one segment (Correa and Dockrell, 2007). Note that in the cases of merging, function words seem to be particularly affected (Roberts, 1992; Ferreiro and Pontecorvo, 2002; Correa and Dockrell, 2007, for observations in Portuguese, Spanish, Italian, and English). Furthermore, in both merging and separation, trochees seem to be a preferred outcome. Moreover, in the given examples of word separation typically morphemes were isolated. However interesting such observations are, unfortunately they are only reported anecdotally or descriptively so far. In a process, which could be termed ‘prosodic bootstrapping’ (Bredel, 2006), prosody-based separation of words may lead over to the third stage of development, which is characterized by morpheme-based borders (e.g., ^∗^<ver^∧^liebt> instead of <verliebt> [in love], Sonja, first grade, reported by Weinhold, 2000, as cited in Bredel, 2006; see Ferreiro and Pontecorvo, 2002 for parallel findings in Spanish). At the following fourth stage, graphemic borders are mainly based on lexical words (e.g., ^∗^<Menschen^∧^weld> instead of <Menschenwelt> [human world], Sonja, first grade, reported by Weinhold, 2000, as cited in Bredel, 2006). At the final stage, graphemic words are identical with syntactic words, which is the guiding principle in the conventionalized German writing system.

With respect to the subsyllabic level, there is evidence that writers are sensitive to graphotactic regularities (for review, see Pacton et al., 2012). In tasks typically exploiting pseudowords, children in first or second grade (or even earlier) demonstrated knowledge of statistical relationships of letter sequences which they were never taught explicitly (Treiman, 1993; Cassar and Treiman, 1997; Pacton et al., 2002).

To conclude, graphemic border marking may be influenced by speech prosody during early phases of children’s writing acquisition. Orthographically inadequate mergers seem to particularly affect function words and both mergers and separations seem to be particularly likely if they result in bisyllabic trochees. A test case for such separations are words which exceed the canonical bisyllabic foot in length, i.e., polysyllabic words. While there are first anecdotal hints on a role of prosodic feet on graphemic border marking, the role of syllabic or subsyllabic regularities remains less clear. After all, no empirical study has systematically investigated the influence of prosodic structure on graphemic border marking in early writing acquisition so far.

### The Present Study

Systematic empirical investigations of speech prosody in writing acquisition are still scarce. In the present study, we analyzed such reflections of speech prosody in German children’s writing acquisition for the first time. To this end, we tested three hypotheses, addressing (1) the omission of between-word graphemic border markers, (2) the addition of word-internal border markers, and (3) the omission of determiners in non-optimal prosodic positions. We examined children in a stage of writing acquisition, which has been claimed to be affected by speech prosody (Bredel, 2006), i.e., 7–8 years of age. The following hypotheses were developed:

(1) Graphemic border markers between lexical words may be omitted or reduced more often, if merging two lexical words leads to one prosodically optimal phonological word.

Prosodically optimal (i.e., canonical) phonological words in German contain at least one trochaic foot consisting of a stressed full syllable and a reduced syllable (Giegerich, 1985; Eisenberg, 2000). An analogous structure is also regarded as graphemically optimal (Evertz and Primus, 2013; Fuhrhop and Peters, 2013). A substantial number of lexical words, however, does not meet this criterion, as they are monosyllabic and thus ‘too short’. This is particularly true for many function words, including prepositions and articles.

We hypothesized that in writing, monosyllabic prepositions should be merged more often with a following definite article than bisyllabic prepositions, as only in the former case – but not in the latter – merging leads to a canonical phonological word. In other words, some children may tend to realize phonological words rather than lexical ones. Specifically, orthographically adequate between-word border markers (i.e., spaces) should be more often reduced or omitted after monosyllabic prepositions as in ^∗^*ín|der Hˊütte*^3^ [in the hut]*, ^∗^mít|den Fréunden* [with the friends] (‘|’ denotes an omitted space between preposition and definite article.) than after bisyllabic prepositions, which already form canonical phonological words on their own as in ^∗^*hínter|dem Félsen* [behind the rock], ^∗^*nében|dem Brúnnen* [next to the fountain]. In fact, merging bisyllabic prepositions with the following article would result in a prosodically non optimal phonological or graphemic word, involving two adjacent unstressed syllables (lapse), one of which cannot be parsed into a binary foot (e.g., [[hín.ter]_F_ dem]]_ω_ [behind the]).

(2) Graphemic border markers may be inserted more often into lexical words, if this leads to prosodically optimal phonological words.

Prosodically optimal phonological words require at least one stressed full syllable and a reduced syllable (Giegerich, 1985; Eisenberg, 2000). A substantial number of lexical words, however, exceed this criterion, as they are polysyllabic, thus being ‘too long’.

We hypothesized that trisyllabic words (here: animal names) should be more often separated by the insertion of orthographically inadequate graphemic border markings, if at least one of the parts forms a prosodically optimal bisyllabic trochaic foot. Moreover, such a graphemic border marking may even more probably be inserted between the two feet of bipedal words (a bisyllabic and a monosyllabic one, e.g., [[pé.li]_F_[kan]_F_]_ω_ [pelican]; [[e.le]_F_[fánt]_F_]_ω_ [elephant]), but be somewhat less likely at borders between a foot and an unparsed syllable (i.e., in monopedal trisyllabic words like [fla[mín.go]_F_]_ω_ [flamingo] or [gi[ráf.fe]_F_]_ω_ [giraffe]), as that unparsed syllable may be too light to build up a foot on its own. More generally, word-internal border markers should be more likely, if they respect borders of prosodic units like foot, syllable, or syllabic constituents, compared to positions which are not motivated by borders of prosodic units. Potentially, the probability of word-internal border markers may reflect the order of the prosodic hierarchy (i.e., foot > syllable > syllabic constituent > others). Examples for such potential orthographically inadequate word-internal markers are given in Example 1 below (‘^∧^’ denoting an added border marking):

**Example 1:** Potential (orthographically inadequate) word-internal borders in the order of the prosodic hierarchy (i.e., with decreasing likelihood of border insertions)

**Table:** 

(a)	foot:	^∗^*Bono*^∧^*bo*^4^, ^∗^*Ele*^∧^*fant* (both bipedal) [bonobo, elephant], *^∗^Fla*^∧^*mingo* (monopedal) [flamingo]
(b)	syllable:	^∗^*Bo*^∧^*no*^∧^*bo* (all three syllables separated) ^∗^*Flamin*^∧^*go* (one syllable separated, foot-internal border)
(c)	syllabic constituent:	^∗^*Fl*^∧^*amingo*, ^∗^*Elefa*^∧^*nt* (syllabic onset or coda separated)
(d)	consonant cluster:	^∗^*F*^∧^*lamingo*, ^∗^*Elefan*^∧^*t* (not prosodically motivated)

(3) Definite articles may be omitted more often in non-optimal prosodic positions.

The optimal prosodic structure of phrases and sentences in German and other languages builds up a strong-weak rhythm (Liberman and Prince, 1977; Vogel et al., 2015). Young children regularize rhythmic structure, by omitting functions words (McGregor and Leonard, 1994; Wijnen et al., 1994; Gerken, 1996; Boyle and Gerken, 1997).

Therefore, we hypothesized that children may again omit function words (here: definite articles) during writing acquisition. In particular, their omissions of definite articles may occur more often, if they lead to regular strong-weak sequences, compared to articles which are already in prosodically optimal positions. Examples for such omission errors are given in Example 2 below (relevant articles underlined):

**Example 2:** Prosodic context favoring omissions (Example 2a) or preservations (Example 2b) of definite articles. (potentially omitted articles scratched, see **Table 1** for English translations)

**Table 1:** Stimulus sentences.

	Stimulus Sentence	English Translation
1	Der_DET1_ Elefánt_N1_ und die_DET2_ Giráffe_N2_ lében in_P_ der_DET3_ H´ütte.	The elephant and the giraffe live in the hut.
2	Der_DET1_ Leopárd_N1_ und der_DET2_ Jáguar_N2_ tóben hínter_P_ dem_DET3_ Félsen.	The leopard and the jaguar romp behind the rock.
3	Das_DET1_ Hermelín_N1_ trinkt aus_P_ der_DET2_ Schále.	The ermine drinks from the dish.
4	Der_DET1_ Pínguin_N1_ und das_DET2_ Krokodíl_N2_ spíelen nében_P_ dem_DET3_ Brúnnen.	The penguin and the crocodile play next to the fountain.
5	Der_DET1_ Márabu_N1_ und der_DET2_ Flamíngo_N2_ spíelen mit_P_ den_DET3_ Fréunden.	The marabou and the flamingo play with the friends.
6	Das_DET1_ K´änguru_N1_ und der_DET2_ Kákadu_N2_ líegen únter_P_ den_DET3_ B´äumen.	The kangaroo und the cockatoo are lying under the trees.
7	Der_DET1_ Papagéi_N1_ und der_DET2_ Pélikan_N2_ éssen auf_P_ der_DET3_ Wíese.	The parrot and the pelican eat on the meadow.
8	Der_DET1_ Bónobo_N1_ und der_DET2_ Kórmoran_N2_ láufen ´über_P_ das_DET3_ Úfer.	The bonobo and the cormorant run across the riverside.

*Relevant words (i.e., those that were entered into the analyses) are indexed for their type of speech: DET = definite article, N = noun, P = preposition. Main stress positions are indicated here for the sake of clarity, but note that they are not indicated in standard German orthography.*


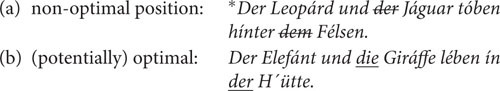


Furthermore, sentence initial positions may be special as they profit from particular phonetic strength (Cho and Keating, 2009) and/or increased attention (Givón, 1988). Therefore, articles in sentence initial positions may be relatively preserved from being omitted.

## Materials and Methods

### Participants

We examined pupils of 4 second grade classes in two regular basic primary schools in North-Rhine Westphalia, Germany. Altogether, 79 children (34 girls, 45 boys) took part. The study was performed in early spring, i.e., after about half of the second school year. Thus, participants were between 7 and 8 years of age. For all participants the language of schooling was exclusively German, for 46 of them, German was also their first language.

Ethical approval for this study has been obtained from the Ethics Committee of the *Deutsche Gesellschaft für Sprachwissenschaft* (DGfS).

### Task and Procedure

Participants were asked to perform a writing to dictation task in a group setting (i.e., the whole class together). After a short introduction by the experimenter, sentences were presented from audio files. Stimuli were repeated as often as requested by at least one pupil, but always as whole sentences.

### Stimuli

The stimulus material consisted of eight sentences^5^. Each sentence contained two (or in one case only one) nominal phrases consisting of animal names preceded by a definite article. Moreover, each sentence ended with a prepositional phrase (e.g., *in der Hˊütte, hinter dem Félsen*), i.e., a preposition (one syllable or bisyllabic trochee), a definite article (one syllable), and a noun (bisyllabic trochee). An overview of all stimulus sentences and their respective English translations is provided in **Table 1**.

The animal names used in the stimulus sentences (total *n* = 15, see **Table 2** for psycholinguistic properties and English translations) were all trisyllabic and had all possible stress patterns – antepenultimate (APU), penultimate (PU), and ultimate (U) stress (e.g., *Pélikan, Flamíngo, Elefánt*). For words with antepenultimate and ultimate stress we assume the same underlying bipedal foot structure consisting of one bisyllabic foot and one foot built up by only one syllable which is mostly heavy (e.g., [[pé.li]_F_[kan]_F_]_ω_; [[e.le]_F_[fánt]_F_]_ω_), while another structure, consisting of one foot and an unfooted syllable, is assumed for words with penultimate stress (e.g., [fla[mín.go]_F_]_ω_) (Domahs et al., 2008, 2013; Janßen and Domahs, 2008; Röttger et al., 2012). Thus, potential word-internal borders motivated by the foot structure are after the second syllable in words with APU and U stress, and after the first syllable in words with PU stress (see Example 1).

**Table 2:** Animal name stimuli.

Item Nr.	Stimulus	Translation	AoA	Freq	Nr. of feet	Maximum possible number of borders			
						Foot	Syll	Syll const	Cons cluster
1	Elefánt	elephant	3,4	13,8	2	1	2	3	1
2	Giráffe	giraffe	3,2	2,6	1	1	2	3	1
3	Leopárd	leopard	5,2	0,4	2	1	2	3	1
4	Jáguar	jaguar	5,9	1,8	2	1	2	3	0
5	Hermelín	ermine	10,8	0,1	2	1	2	5	0
6	Pínguin	penguin	3,5	29,6	2	1	2	4	0
7	Krokodíl	crocodile	3,6	9,5	2	1	2	4	1
8	Márabu	marabou	11,0	0,0	2	1	2	3	0
9	Flamíngo	flamingo	6,6	0,9	1	1	2	4	1
10	K´änguru	kangaroo	4,7	7,8	2	1	2	4	0
11	Kákadu	cockatoo	7,0	6,1	2	1	2	3	0
12	Papagéi	parrot	4,0	12,6	2	1	2	3	1
13	Pélikan	pelican	6,8	4,2	2	1	2	3	0
14	Bónobo	bonobo	11,7	0,4	2	1	2	3	0
15	Kórmoran	cormorant	11,6	0,3	2	1	2	5	0

*AoA, Age of Acquisition (mean retrospective ratings from 17 adult participants in years); Freq = word frequency per million from Childlex.db database for the age range from 6 to 12 years (Schroeder et al., 2014); Syll: borders between syllables; Syll const = borders between syllabic constituents, i.e., onset, nucleus, and coda; Cons cluster = borders between consonants within a syllable onset or coda. Note that main stress position is not indicated in standard German orthography.*

Some of the animal names used are quite familiar to children aged seven or eight (e.g., *Elefant, Pinguin*, and *Papagei* [elephant, penguin, parrot]), others are rather infrequent and typically acquired late (see **Table 2** for age of acquisition values), such that it seems likely that they were unfamiliar to most of the participants (e.g., *Hermelin, Marabu*, and *Kormoran* [ermine, marabou, cormorant]).

### Coding and Scoring Procedures

(1) Border marking between prepositions and definite articles

**FIGURE 1:**
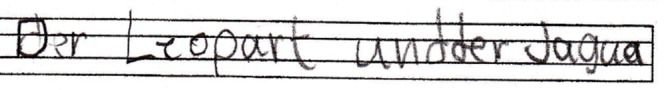
**Example for challenges in objective measurements of inter-letter spacing.** Target: <Der Leopard und der Jaguar> [The leopard and the jaguar]. Should the smallest horizontal or the smallest absolute distance be measured? For instance, does the distance between <L> and <e> in <Leopart> (Leopard) refer to the upper or the lower part of the <e>? When measuring the distance between <r> and <t>, should the vertical line (i.e., the head in the sense of Primus, 2004) or the small horizontal line (i.e., the coda) of the <t> be considered? What about vertical lines, which are actually inclined as in the <t>? When relativizing inter-letter spaces with the size of letters to make them comparable across different words or different children, which letters should one chose, given that there are apparently smaller ones (e.g., <e>) as well as larger ones (e.g., <p>) within the same word. Even though word-internal inter-letter spaces may be different at different positions (as in this example), they may still all be categorically perceived as ‘no border’, especially given the much larger distance between the lexical words <Der>, <Leopart>, and <und>. (However, note the reduced inter-word distance between <und> and <der>).

First, we analyzed potential mergers of preposition and definite article. To this end, four independent raters performed graded perceptual judgments of graphemic borders between prepositions and articles. On a five-point scale they were asked to judge, whether a border was clearly present (+2), clearly absent (-2) or something in between (+1, 0, or -1). Perceptual judgments were used rather than objective measures, as borders can be marked by a number of different means including spacing, capitalization, hyphenation, punctuation, and combinations thereof (Lamme, 1984; Rowe and Harste, 1986; Martens and Goodman, 1996). Moreover, the main type of border markings, i.e., spacing, is related in non-trivial ways to the (fluctuating) size of letters and inter-letter distances (see **Figure 1**). Furthermore, it is often not clear, from which points a distance between two letters should be measured and how the inter-letter distance should be relativized with the absolute letter size (see **Figure 1**).

Finally, as some definite articles (e.g., *der, den, dem*) show more resemblance to typical word-final reduced syllables than others (e.g., *das*), the former may be more prone to merging with a preceding preposition than the latter. Therefore, we distinguished between both types of articles.

(2) Word-internal border marking within animal names

Secondly, we examined orthographically inadequate graphemic border markers inserted into animal names. Four independent raters, different from the ones before, rated transitions between all consecutive letters for potential word-internal borders within the animal names on a three-point scale from 0 (clearly absent) over 0.5 (uncertain) to 1 (clearly present). Thus, in an ideal case, all ratings should be zero, given that in German orthography no word-internal borders should be marked. Ratings were classified for type of prosodic border (feet, syllables, syllabic constituents, or consonant clusters; see Example 1), averaged across raters per category, and corrected for the maximum number of borders possible for each category (see **Table 2**).

Note that border insertions separating feet and syllables cannot be clearly differentiated. All foot-based border insertions coincidently separate syllables, too, while border insertions separating all three syllables also separate a foot. Yet, the classification chosen in Example 1 is the most conservative with respect to the prosodic hierarchy, because only the clearest cases of a foot-based border are classified as foot-based, while the more ambiguous cases (all three syllables separated) are classified as syllable-based. Therefore, graphemic markings at the border of feet were only analyzed as foot-based, although this is at the same time also a border between syllables (e.g., ^∗^<*Kormo*^∧^*ran*> [cormorant]). On the other hand, graphemic markings of both syllabic borders of a word were only counted as syllabic, even though one of them is at the same time a foot-based border (e.g., ^∗^<*Pa*^∧^*pa*^∧^*gei*> [parrot], where the latter border is both foot- and syllable-based).

Responses, in which the syllabic structure was changed by a child (e.g., ^∗^<*groKdl*> instead of <*Krokodil*> [crocodile]), were excluded from analyses, whereas responses with orthographic errors preserving structure (e.g., ^∗^<*Hermolin*> instead of <*Hermelin*> [ermine]) were included. Errors leading to consonant omissions within clusters (e.g., ^∗^<*Elefat*> instead of <*Elefant*> [elephant]) or altered vowel length marking were treated as ‘preserving structure’ and included.

(3) Omissions of definite articles in prosodically non-optimal positions

Thirdly, we looked for omissions of definite articles in non-optimal prosodic positions. As article omissions were clearly detectable, only one rater coded them.

Non-optimal and (potentially) optimal positions were defined as in Example 2. Note that it is not always straightforward to define prosodically optimal positions. Some positions can be clearly identified as **non**-optimal, as there is an even number of syllables between two syllables bearing main stress (see Example 2a), making a strong-weak rhythm logically impossible. However, although an odd number of syllables between two main stress positions potentially allows for a regular rhythm, this regular rhythm may or may not be realized phonetically. Considering Example 2b, to fulfill the Rhythm Rule, the first underlined article should be realized (relatively) strong, whereas the second underlined article should be realized weak. In principle, function words may adopt the strength of their expression to their rhythmic position (Selkirk, 1996; Vogel et al., 2015). Whether or not this is actually the case in stimuli as used in our study is a question of empirical phonetic analysis, which is beyond the scope of this paper. Nevertheless, we expect that, in general, potentially optimal positions are preferred compared to non-optimal positions.

In addition, we coded whether articles appeared in sentence-initial or medial position, assuming that the former may be more insusceptible to omissions. Finally, we analyzed phonetic salience of definite articles in the stimulus recordings as salience may be related to the number of omissions. To this end, mean pitch, duration, and intensity of all definite articles were measured using *Praat* (Boersma and Weenink, 2013).

### Statistical Analyses

Given that perceived border markings between prepositions and definite articles were rated on a five-point scale and most borders were clearly marked, the resulting data yielded a non-normal, negatively skewed distribution. Therefore, we performed a non-parametric Wilcoxon signed rank test for repeated measures (within subject comparisons) with one-tailed exact analysis to compare potential border markings after bisyllabic vs. monosyllabic prepositions.

Concerning word-internal border markings within animal names, we used linear mixed effects models with mean corrected perceptual scores as dependent variable and *item* and *participant* as random factors as well as *intercept, type of border* (foot, syllable, syllabic constituent, and consonant cluster), *foot structure* (bipedal vs. monopedal), an interaction of *type of border × foot structure*, and *linguistic background* (German as L1 vs. L2) as fixed factors, as well as *age of acquisition* and *word frequency* as covariates. Starting with the most complex model, we removed all terms which did not contribute significantly to the variance explained in a backward procedure. If random effects did not account for a meaningful amount of variance (i.e., if the residual variance was larger than the random effect variance estimates), then the random effects were eliminated from the model and a standard model was fitted.

With respect to article omissions, their occurrence in prosodically optimal vs. non-optimal positions was compared using the non-parametric Mann–Whitney *U*-test for unrelated samples. To compare acoustic cues (i.e., pitch, duration, and intensity) of articles in prosodically optimal vs. non-optimal positions, two-tailed unpaired *t*-tests were performed. Finally, Spearman’s rank correlations were calculated between articles’ acoustic cues and number of omissions.

## Results

**FIGURE 2:**
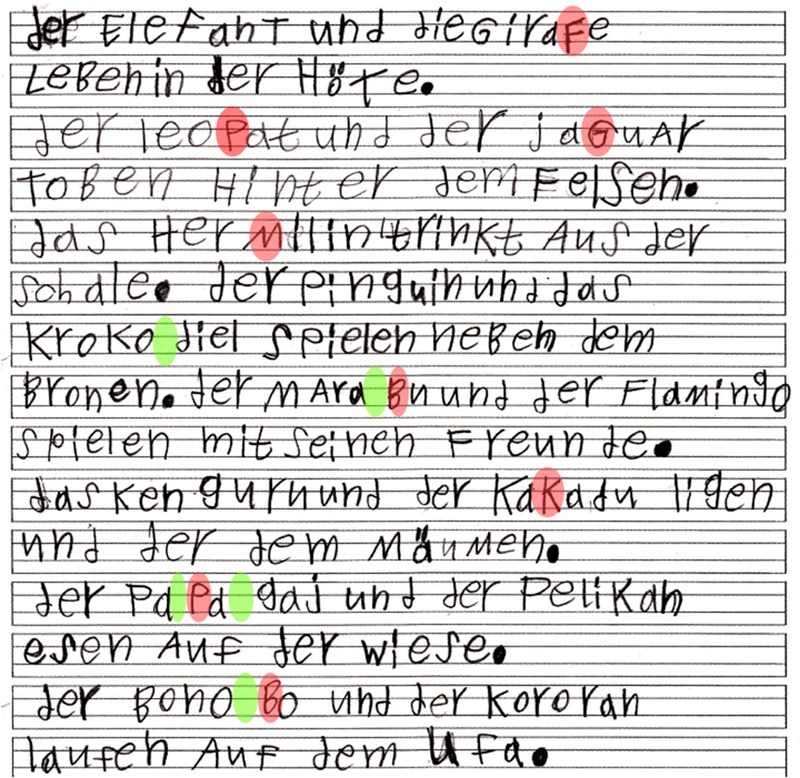
**Example of a dictation (participant 37; 7 years old; L1 Arabic).** Some graphemic border phenomena relevant to the present analyses can be illustrated: Potential border marking by (word-internal) capitalization (red markers): <GiraFe> (Giraffe, line 1), <jaGuar> (line 3), <HerMilin> (Hermelin, line 5), <MAraBu> (line 8), <KaKadu> (line 10), <PaPagaj> (Papagei, line 12), <BonoBo> (line 14). Potential word-internal border marking by spacing (green markers): <KroKo^∧^diel> (Krokodil, foot-based, line 7), <Mara^∧^Bu> (foot-based, line 8), <Pa^∧^Pa^∧^gaj> (Papagei, leaving a smaller space between the first and second syllable – i.e., syllable based border – and a larger space between the second and third syllable – i.e., foot-based border, line 12), <Bono^∧^Bo> (foot-based, line 14). Note that capitalization and spacing may co-occur (e.g., <Mara^∧^Bu>, <Pa^∧^Pa^∧^gaj>, and <Bono^∧^Bo >), probably leading to increased salience of perceived graphemic borders. No definite article was omitted, but one article (<den>) was replaced by a possessive pronoun (<seinen>, line 9). Moreover, some phenomena can be observed which are related but not specifically subject of the present analyses: Potential merging of definite article and noun: <die> and <GiraFe> (Giraffe, line 1). Insertion of an inadequate border within a preposition, resulting in three existing function words: <und der dem> (instead of <unter dem>, line 11). For English translations and word type annotations see **Table 1**.

All participants were well able to perform the task. A particularly illustrative example is given in **Figure 2**.

(1) Border marking between prepositions and definite articles

The overwhelming majority of the borders between prepositions and definite articles could be clearly identified, meaning that they were realized correctly. Numerically, borders were perceived with increasing certainty in the predicted order: trochaic bisyllabic prepositions followed by definite article *das* > trochaic bisyllabic prepositions followed by definite article *dem* or *den* > monosyllabic preposition followed by definite article *der* or *den* (grand mean scores over four raters and all items per type [standard deviations]: 1.77 [0.74] > 1.64 [0.77] > 1.60 [0.84], respectively). However, these differences did not approach statistical significance (Wilcoxon signed rank test, one-tailed exact *p* ≥ 0.425). Given that a score of +1 indicated “quite certainly” and a score of +2 “absolutely certainly” perceived borders, results were obviously very near to ceiling here.

(2) Word-internal border marking within animal names

**FIGURE 3:**
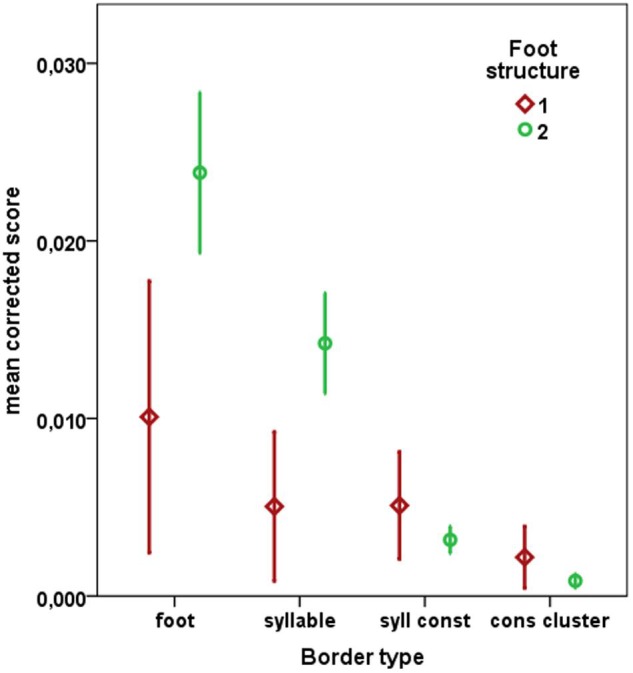
**Word-internal graphemic borders within trisyllabic animal names as a function of type of border and foot structure.** Border types: foot = foot-based, syllable = syllable-based, syll const = syllabic constituent, cons cluster = consonant cluster (see **Table 2**); Foot structure: 1 foot = trisyllabic monopedal words with one binary trochaic foot and one unparsed syllable, main stress position on penultimate syllable (PU), 2 feet = trisyllabic bipedal words with a final monosyllabic foot and a preceding binary foot, main stress position on ultimate (U) or antepenultimate (APU) syllable (see Stimuli section). Note that the maximum possible corrected score would be 1, meaning that all four raters had perceived borders in all possible instances with greatest certainty. On the other hand, the mean corrected score for orthographically correct realizations would be 0, as no single border marking should occur within lexical/graphemic words. Error bars indicate 95% confidence intervals.

In general, word-internal border markings within trisyllabic animal names occurred only rarely. Still, in dictations from 58 of the 79 children at least one of the raters detected at least one word-internal border within an animal name. Crucially, when occurring, word-internal border markings were influenced by prosodic structure (see **Figure 3**): Most of them were produced at borders of feet (in dictations from 43 children, at least one rater detected at least one border of this type).

Beyond a constant for intercept, the final linear mixed effects model on mean corrected perceptual scores as dependent variable contained only *type of border* (foot, syllable, syllabic constituent, and consonant cluster) and the interaction of *type of border × foot structure* as fixed factors (*F* ≥ 5.29, *p* < 0.001). Numerically, borders occurred roughly in the order of the prosodic hierarchy, i.e., foot > syllable > syllabic constituent > consonant cluster (see **Figure 3**). *Post hoc* contrasts revealed significant differences between foot vs. syllable, foot vs. syllabic constituent, foot vs. consonant cluster, and syllable vs. syllabic constituent (all Bonferroni adjusted *p* ≤ 0.026). All other contrasts failed to reach statistical significance.

Models containing the fixed factors *foot structure* (bipedal vs. monopedal), or *linguistic background* (German as L1 vs. L2), and the covariates *age of acquisition* or *word frequency* were disregarded as they did not explain significant additional variance. The random factors *subject* or *item* did not account for meaningful amounts of variance and were also eliminated from the model.

(3) Omissions of definite articles in prosodically non-optimal positions

**FIGURE 4:**
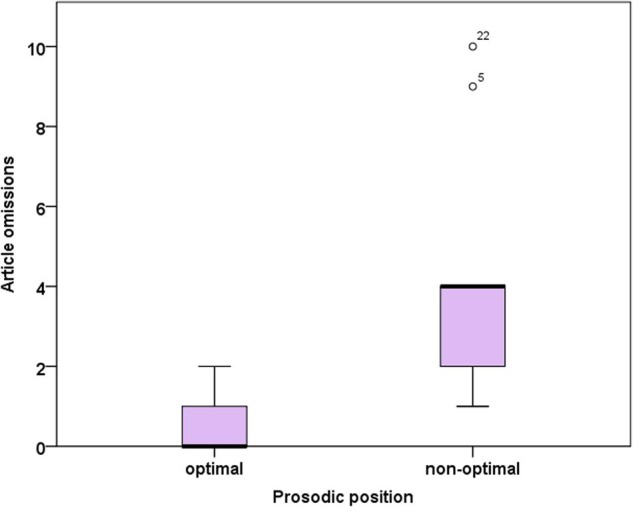
**Omissions of definite articles as a function of prosodic environment.** optimal = potentially consistent strong-weak rhythm, non-optimal: consistent strong-weak-rhythm impossible due to an even number of syllables in between two stressed syllables (see Example 2).

In 49 out of 1817 cases (2.7%), a definite article was omitted. Interestingly, we observed more omissions of the definite article in non-optimal prosodic positions (where only the omission of a syllable allows for a consistent strong-weak rhythm, see Example 2a) compared to prosodically optimal positions (where a consistent strong-weak rhythm is potentially possible without syllable omission, see Example 2b, see **Figure 4**). This difference was statistically significant (Mann–Whitney *U* = 116.0, *p* < 0.001). Given that articles in sentence-initial position may benefit from phonetic strengthening and/or increased attention and no sentence-initial article was classified as occurring in a prosodically non-optimal position, results on article omissions may be biased by sentence-initial articles. Therefore, we performed the same analysis without sentence-initial articles. Again, significantly more article omissions occurred in non-optimal compared to optimal prosodic environments (Mann–Whitney *U* = 37.5, *p* = 0.008).

**FIGURE 5:**
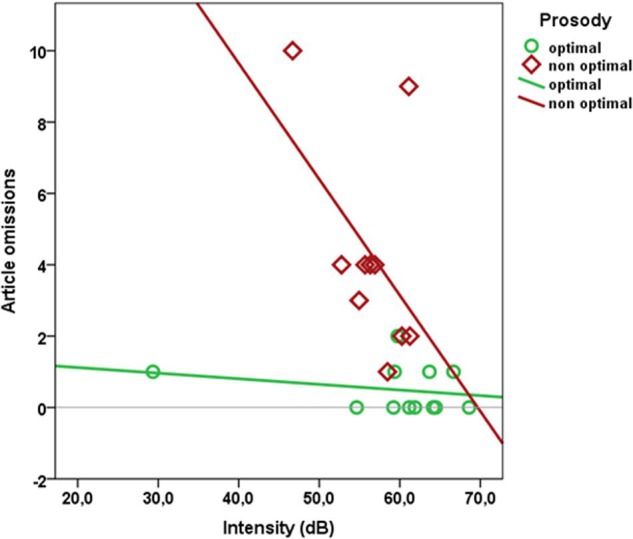
**Number of article omissions as a function of mean intensity of simuli for optimal and non-optimal prosodic environments**.

Were article omissions related to phonetic features of the auditory stimulus? Mean pitch, duration, or intensity of auditorily presented articles did not differ significantly between articles in optimal vs. non-optimal positions (see **Table 3**). However, there was a significant negative correlation between mean intensity and number of omissions (Spearman’s rank correlation *r*_S_ = -0.49, *p* = 0.017; see **Figure 5**). As can be seen in **Figure 5**, this correlation was mainly driven by articles in prosodically non-optimal environments. The number of omissions did not correlate with mean pitch or duration of the article (*p* ≥ 0.15).

**Table 3:** Phonetic cues (means and standard deviations) for definite articles realized in prosodically optimal vs. non-optimal environments (within the stimulus material).

	Prosodic position		
	Optimal	Non-optimal	Significance
Pitch (Hz)	238.4 (76.7)	209.1 (32.1)	*t* = –1.13, *p* = 0.27
Duration (ms)	154.2 (29.9)	171.4 (50.5)	*t* = 0.99, *p* = 0.33
Intensity (dB)	59.4 (10.2)	56.4 (4.4)	*t* = –0.85, *p* = 0.40

*The Significance column reports values from two-tailed unpaired *t*-tests.*

## Discussion

The present study aimed at finding reflections of speech prosody in children’s writing. In a sample of second graders’ texts handwritten from dictation we found

(1) no significant evidence for omission or reduction of graphemic border markers between monosyllabic prepositions and subsequent definite articles,
(2) evidence for insertion of graphemic borders into trisyllabic words (animal names), in the order predicted by the prosodic hierarchy (foot > syllable > subsyllabic constituents > consonants within clusters) and modulated by foot structure (i.e., more probable for bipedal compared to monopedal words). The occurrence of inadequate borders was not modulated by the word’s age of acquisition (AoA) or frequency or children’s language status (German as L1 or L2).
(3) evidence for omissions of definite articles in non-optimal prosodic positions and a significant negative correlation between an article’s mean stimulus intensity and its number of omissions, but no other detectable phonetic influence.

The fact that we did not observe significantly reduced border markings between monosyllabic prepositions and definite articles as compared to bisyllabic prepositions and articles can plausibly be attributed to a ceiling effect. Indeed, the overwhelming majority of the borders between prepositions and definite articles were clearly realized. In general, second graders may be already familiar with prepositions and articles as graphemic words, preventing them from writing (merged) phonological words. This knowledge may get lost again in acquired disorders of writing, such as in a patient with surface dysgraphia, who frequently omitted graphemic borders between lexical words which combine to phonological words (e.g., ^∗^<LASDAS> instead of <lass das> [drop it], Bormann et al., 2009).

Word-internal borders were most likely introduced at positions, where a canonical bisyllabic trochaic foot or a syllable were separated (see **Figure 3**). These border insertions may reflect premature hypotheses on the graphemic marking of spoken prosodic cues. Crucially, the most important prosodic unit guiding word-internal graphemic border markings was the prosodic foot. Note that border insertions which isolated all three syllables of a target word were classified as syllable-based, even though in such cases one of the two borders also separated a foot, so that foot-based border insertions may even be somewhat undervalued. In general, our results are consistent with the assumption of a prosodic stage in the acquisition of graphemic border marking as proposed by Bredel (2006). However, the fact that word-internal border markings occurred only rarely in our sample may suggest that most of the pupils examined in the present study had already progressed into later stages of development.

At first glance, it may seem surprising that we did not observe any influence of the AoA or frequency of target words on border insertions, even for those words which were probably not yet acquired with 8 years of age. In principle, lexical familiarity may influence or complement prosodic processing (Boyle and Gerken, 1997) as well as writing (Bonin et al., 2001; Perret et al., 2014). However, it should be pointed out that the present AoA values were rated without any mention of written language. In consequence, ratings most likely refer to spoken rather than written language. Therefore, even words which were highly familiar to the participants in spoken language were probably unfamiliar to them in written language, making them prone to unconventional spellings (‘errors’). In the same vein, written word forms of our target items can be regarded as equally unfamiliar to children with German as L2 or L1, even though the spoken word forms may be more familiar to native speakers of German. Yet, the principle how to spell these word forms has to be acquired likewise for all children irrespective of their individual linguistic background.

In principle, border insertions within written polysyllabic words may have different underlying causes. First, they may directly reflect the prosodic structure of spoken input or of children’s mental phonological representations (‘phonographic account’). Second, they may be manifestations of children’s intermediate hypotheses on the inherent graphemic structure of written German (‘graphemic account’). Third, they may bring to surface units of graphomotor execution in children’s handwriting (‘motor account’). These three accounts are not mutually exclusive and the present study was not designed to disentangle them. However, given the early stage of writing acquisition (at which writing is still strongly depended on spoken language, e.g., Binamé and Poncelet, 2016) and the nature of the task (writing to dictation), to us a phonographic explanation seems more likely than a graphemic account. Following the phonographic explanation, children would have to discover regularities, which guide the conversion of spoken (suprasegmental) prosodic cues to written language in addition to (segmental) phoneme-grapheme conversion. Yet, this does not rule out a motor production component also being involved. In fact, it has been shown that features of graphomotor execution in writing can be related to preceding cognitive stages of spelling (Lochy et al., 2002, 2004). Importantly, such influences may reflect chunking into processing units like syllables or morphemes in adult writers (Weingarten et al., 2004; Will et al., 2006; Kandel et al., 2012, 2013; Bertram et al., 2015) as well as in writing acquisition (Kandel and Perret, 2015). When distinguishable, orthographic syllables seem to be more relevant processing units than phonological syllables (Lambert et al., 2015). However, even though some influence of chunking units at the motor execution stage of writing cannot be excluded and does even seem a plausible cause of space insertions, there are some arguments which relativize the explanatory power of the motor account for the present results: First, it would seem a remarkable coincidence that chunking units in motor execution are largely identical to prosodic units, if they were completely independent. Moreover, second graders should be (and are) well aware of the fact that the result of their writing should reflect graphemic/orthographic rather than motor units. Thus, features of motor production may affect gradual (sub-threshold) but merely categorical distances between letters (categorical meaning that they distinctively mark a border). Note that our dependent variable was graded perceptual ratings, which reflect both gradual and categorical distances. Finally, the motor account can only explain (gradual) border marking by distance, but not by capitalization, which also occurred in our data (see **Figure 2**). In sum, we don’t think that a motor account can fully explain the present data. Rather, a phonographic account, with some possible influences of graphemic knowledge and graphomotor execution seems most plausible.

As we have argued in the Methods section, it is not trivial to find objective measures of graphemic borders. Therefore, we have chosen a perceptual approach in our analyses, circumventing these difficulties. Given that the raters were naïve with respect to the aims of our study, we do not assume any hypothesis-driven influences on their perceptual ratings. However, as all raters were competent speakers of German, we cannot fully exclude that their (explicit or implicit) prosodic knowledge may have modulated their ratings. Still, we don’t see how an artifact of rating could explain that borders were observed in some children but not in others. A developmental approach, on the other hand, can smoothly explain this finding with the assumption that only some children were in a prosodic stage of writing acquisition at the time of our examination. Yet, we cannot disentangle subject-based from rater-based effects with absolute certainty. In the future, studies employing more objective measures (e.g., registration and analysis of motor execution of writing) should complement the present results.

The omission of definite articles parallels the observation that function words in non-optimal prosodic positions are often omitted in repetition tasks by much younger children (McGregor and Leonard, 1994; Wijnen et al., 1994; Gerken, 1996; Boyle and Gerken, 1997). Two probably intertwined explanations can be given for the present observation. First, it may be that omissions reflect some phonetic feature(s) of the stimulus material (‘phonetic account’). Indeed, it has been shown that function words may adopt their phonetic characteristics to their respective rhythmic position (Selkirk, 1996; Vogel et al., 2015). In apparent support of the phonetic account, there was a significant negative correlation between stimulus intensity and the number of article omissions. However, **Figure 5** reveals that this correlation was exclusively driven by determiners in non-optimal positions, while there was no relationship between intensity (or other relevant phonetic cues) and omissions of articles in (potentially) optimal positions, reducing the explanatory power of the phonetic account. Moreover, even articles in non-optimal positions were realized by a large majority of children (the most affected article was still correctly realized by 69 out of 79 children), making a purely phonetic account even less compelling.

Second, given that the task (writing to dictation of whole sentences) includes a high load on working memory, children may resort to the support of more abstract – phonological – representations (‘phonological account’). It is well known that prosodic structure influences storage in short term in memory (e.g., Reeves et al., 2000). Thus, children’s representations may be more robust for prosodically optimal positions, where articles are more deeply integrated into prosodic structure compared to non-optimal positions (Gerken, 1996). Interestingly, it has been shown, that in adults the written production of determiners (e.g., writing rate and pauses) is influenced by phonological properties (e.g., syllabic length, phoneme-to-grapheme consistency) of the following noun (Maggio et al., 2015). This may reflect similar underlying effects of parallel or incremental processing at the orthographic and motor levels of writing. In the light of these arguments, we think that the systematic nature of article omissions in our study reflects characteristics of phonological representations involved. Note that the phonological account does not exclude the existence of phonetic correlates of article omissions. Rather, it states that more abstract mental representations are involved and omissions are not only due to more peripheral perceptual processes.

Altogether, our results provide first empirical evidence from children acquiring written German for prosodic influences on writing in a language with scarce graphemic marking of prosody.

## Author Contributions

All authors conceived the study and its design. KB performed data collection. KB, FD, and UD performed data processing and statistical analysis and interpreted the data. FD drafted the manuscript. All authors read and approved the final manuscript.

## Conflict of Interest Statement

The authors declare that the research was conducted in the absence of any commercial or financial relationships that could be construed as a potential conflict of interest.

## Footnotes

1 For German, it has been argued that phonological words are not necessarily isomorphic with lexical, morphological, or syntactic words. This holds especially for derived words including certain types of suffixes, prefixed words, compounds as well as for lexical words with clitizised function words (Wiese, 2000; Fuhrhop, 2008). For the purpose of the present study, in most cases we will not distinguish between lexical and morphological or syntactic words, only using the shorthand term *lexical word* as opposed to the phonological or prosodic word.

2 Throughout the paper, English translations will be provided in squared brackets.

3 Stress markers are not used in standard German orthography.

4 Note that, in German, Bónobo is stressed on the first syllable.

5 Originally, twelve sentences were prepared for dictation. However, as in one class it was only possible to perform the first eight sentences within one lesson, the task was reduced to those sentences for the following classes as well.
